# Novel Research on Selected Mechanical and Environmental Properties of the Polyurethane-Based P3HB Nanobiocomposites

**DOI:** 10.3390/ma18112664

**Published:** 2025-06-05

**Authors:** Iwona Zarzyka, Beata Krzykowska, Karol Hęclik, Wiesław Frącz, Grzegorz Janowski, Łukasz Bąk, Tomasz Klepka, Jarosław Bieniaś, Monika Ostapiuk, Aneta Tor-Świątek, Magda Droździel-Jurkiewicz, Joanna Paciorek-Sadowska, Marcin Borowicz, Adam Tomczyk, Anna Falkowska, Michał Kuciej

**Affiliations:** 1 Department of Organic Chemistry, Faculty of Chemistry, Rzeszów University of Technology, Powstańców Warszawy 6, 35-959 Rzeszów, Poland; b.krzykowska@prz.edu.pl; 2 Department of Biotechnology and Bioinformatic, Rzeszów University of Technology, Powstańców Warszawy 6, 35-959 Rzeszów, Poland; kheclik@prz.edu.pl; 3 Department of Materials Forming and Processing, The Faculty of Mechanical Engineering and Aeronautics, Rzeszow University of Technology, al. Powstańców Warszawy 12, 35-959 Rzeszów, Poland; wf@prz.edu.pl (W.F.); gjan@prz.edu.pl (G.J.); lbak@prz.edu.pl (Ł.B.); 4 Department of Technology and Polymer Processing, Faculty Mechanical Engineering, Lublin University of Technology, Nadbystrzycka 36, 20-618 Lublin, Poland; t.klepka@pollub.pl (T.K.); a.tor@pollub.pl (A.T.-Ś.); 5 Department of Materials Engineering, Faculty Mechanical Engineering, Lublin University of Technology, Nadbystrzycka 36, 20-618 Lublin, Poland; j.bienias@pollub.pl (J.B.); m.ostapiuk@pollub.pl (M.O.); m.drozdziel@pollub.pl (M.D.-J.); 6 Department of Chemistry & Technology Polyurethanes, Faculty of Materials Engineering, Kazimierz Wielki University, JK Chodkiewicza Street 30, 85-064 Bydgoszcz, Poland; sadowska@ukw.edu.pl (J.P.-S.); m.borowicz@ukw.edu.pl (M.B.); 7 Department of Mechanics and Applied Computer Science, Faculty of Mechanical Engineering, Bialystok University of Technology, 45C Wiejska Str., 15-351 Bialystok, Poland; a.tomczyk@pb.edu.pl (A.T.); a.falkowska@pb.edu.pl (A.F.); m.kuciej@pb.edu.pl (M.K.)

**Keywords:** polyhydroxyalkanoates, polyurethane, montmorillonite, polymer nanobiocomposites, biodegradability, mechanical properties, thermal stability

## Abstract

This study focused on hybrid nanobiocomposite polymers produced with the use of poly(3-hydroxybutyrate), P3HB and aliphatic polyurethane (PU) as a matrix, including variable quantities of organomodified montmorillonite (Cloisite^®^30B). Mechanical, thermal, and biodegradability tests were conducted to evaluate their properties. The nanobiocomposites were tested using monotonic tensile tests, which revealed that the addition of PU and organomodified montmorillonite reduced the stiffness and strain at break compared to native P3HB. The material’s yield strength was higher for P3HB, while the PU-modified composites exhibited lower stiffness and increased ductility, especially with lower amounts of clay. Scanning electron microscopy (SEM) images showed that cracks in the samples propagated more rapidly as the clay content increased. The bending test showed that the P3HB–PU composites and the nanobiocomposites exhibited lower bending strength and elongation at break compared to pure polyester. However, the composites with lower clay content showed better performance, suggesting that clay promotes ductility to some extent. The Charpy impact tests indicated an increase in impact strength for the composites with the addition of PU and montmorillonite, especially for the samples with 1 wt.% clay. Biodegradability testing showed that P3HB has a biodegradability of 63.21%. However, the addition of clay reduced biodegradability, with a notable decrease as the clay content increased. The biodegradation of composites with 1 and 2% by mass clay was higher than that of P3HB. Thermal analysis indicates an improvement in the thermal stability of the nanomaterials, with the 1% by mass clay sample showing the highest decomposition onset temperature (263 °C). Overall, the study demonstrated that the presence of PU and montmorillonite moderated the mechanical and thermal properties and biodegradation of P3HB, with the optimal performance observed in the composites with 1% by mass clay.

## 1. Introduction

The increasing environmental concerns related to the excessive use of petroleum-based plastics and their persistence in natural ecosystems have prompted a significant shift towards the development of biodegradable and sustainable materials. In this context, biopolymers, particularly polyhydroxyalkanoates (PHAs), have gained considerable attention as alternatives to conventional plastics due to their inherent biodegradability and eco-friendly characteristics. Among the various PHAs, poly-3-hydroxybutyrate (P3HB) stands out as a promising candidate due to its favorable mechanical properties, high biodegradability, and its ability to be produced by microorganisms from renewable resources [1,2,3]. However, despite these advantages, the practical applications of P3HB are limited by its relatively low mechanical strength, poor flexibility, and high brittleness, which restrict its use in applications requiring higher durability and toughness [4,5,6].

To overcome these limitations, researchers have explored various strategies to modify the properties of P3HB, one of which involves blending it with other polymers or incorporating nanomaterials. Blends of P3HB are produced with other polymers, both synthetic, including polylactide (PLA), poly(vinyl alcohol) (PVAL), poly(vinyl acetate) (PVAc), poly(methyl methacrylate) (PMMA), poly(cyclohexyl methacrylate) PCHMA, polycaprolactone (PCL), and natural (chitosan, starch, PHAs). The polymer most often used to create blends with P3HB is PLA [7,8], which showed a decreasing the melting temperature and reducing crystallinity of the P3HB blends and improved flexibility and impact strength, while maintaining biodegradability. The mixing of PVA with P3HB causes a decreasing in crystallinity and increased susceptibility to biodegradation [9]. The introduction of P(VAc-co-VA) to P3HB improves the mechanical properties of the composition and inhibits crystallization [10]. P3HB/PMMA blends were produced in the form of single-phase amorphous glass materials, and their glass transition temperature value depends on their composition. The P3HB crystallization was delayed. In turn, P3HB did not show miscibility with PCHMA and formed a two-phase system [11]. The addition of PCL improved the mechanical properties of P3HB and reduced its crystallinity degree, while the thermal properties of the material were not impaired [12]. Blending P3HB with chitosan results in decreased crystallinity and lower melting and glass transition temperatures of P3HB [13,14]. Blends of P3HB and cellulose show an increase in tensile strength [15]. Blends of P3HB and copolymer P(3HB-co-3HV) show reduced glass transition and melting temperature and decreased crystallinity [16].

It is commonly known that polyurethanes (PUs) are versatile synthetic polymers with excellent flexibility, toughness, and high impact resistance. PU can significantly improve the mechanical properties of P3HB by enhancing its flexibility and toughness, making it a suitable candidate for producing biocomposites with superior performance [17,18,19,20,21,22,23].

Analysis of data from the literature showed that polyurethanes have not been used to modify the properties of native P3HB by preparing binary compositions, except for the work of the authors [24,25,26]. Frone [21] moderated the properties of P3HB using thermoplastic PU, with biodegradable segments of PCL or its copolymer with poly(butyl adipate) (PBA) as a soft segment, in the presence of microfibrillated cellulose, improving the melt processability.

Some studies have explored the use of P3HB to modify polyurethane properties. For example, Saha et al. [22] synthesized polyurethane (PU) from castor oil and a P3HB-based diol using hexamethylene diisocyanate (HDI). Incorporating P3HB notably enhanced the tensile strength, as well as increased the stiffness and crystallinity of the resulting PUs, compared to those derived solely from castor oil.

In contrast, polyurethanes have sometimes been used to modify P3HB copolymers, e.g., PHBV. Wang et al. [18] prepared polymer compositions of PHBV and PU with thermoplastic character (TPU) by mixing in a molten state. TPU addition led to a reduction in crystallinity, an increase of 5 °C in the initial degradation temperature, and a 225% rise in elongation at break versus pure PHBV.

Panaitescu et al. [20] modified the properties of PHBV using two PU elastomers containing PCL or PCL/PBA as the soft segment. The presence of PU resulted in the reduction of the crystallization temperature of PHBV and the degree of crystallinity.

However, PU alone does not fully address the challenges related to the stiffness and mechanical strength of the composite. This has led to the incorporation of nanofillers, such as montmorillonite (MMT), to further enhance the composite properties [27,28,29,30].

Montmorillonite, a naturally occurring clay mineral, is an ideal candidate for reinforcing polymer matrices, owing to its extensive surface area, layered structure, and excellent dispersion ability [31,32,33]. When organically modified, montmorillonite can form strong interactions with the polymer matrix, improving the mechanical strength, thermal behavior, and barrier performance of the final composites. The montmorillonite addition can enhance the dispersion of the polymer chains, strengthen the material, and increase its resistance to heat-induced degradation [34,35,36,37]. Moreover, the presence of montmorillonite in biocomposites has been shown to maintain or even improve the biodegradability of the material, making it an attractive option for developing environmentally friendly alternatives to traditional plastic [38,39,40].

The incorporation of PU and montmorillonite into the P3HB matrix has the potential to address the mechanical and thermal limitations of P3HB while preserving or enhancing its biodegradability. However, the consequences of these modifications for the composite’s mechanical performance, thermal durability, and biodegradability, are still not fully understood. Therefore, it is essential to study the effect of PU and montmorillonite on the performance of P3HB, particularly in relation to its suitability for sustainable and biodegradable applications.

In their earlier work [25], the authors prepared P3HB polymer blends using polyurethanes (PU) based on 1,6-hexamethylene diisocyanate (HDI) and polypropylene glycols (PPG) with molar masses of 400 g/mol and 1000 g/mol. Polyurethanes were incorporated to modify the properties of P3HB at concentrations of 5, 10, 15, and 20 wt.%. The blends exhibiting the best thermal and mechanical performance contained 10 wt.% of HDI-based PU combined with PPG400.

This study aims to test the properties of hybrid nanobiocomposites based on P3HB, polyurethane based on HDI and PPG400, and varying amounts of organically modified montmorillonite (Cloisite^®^30B). A systematic analysis of the mechanical, thermal, and biodegradability characteristics of these nanobiocomposites was conducted to assess how PU and montmorillonite affect P3HB’s behavior. The research investigates how the incorporation of PU and montmorillonite affects the tensile properties, thermal stability, and biodegradability of the composites, with the ultimate goal of developing a material that combines enhanced mechanical performance with environmental sustainability.

P3HB, polyurethanes, and organically modified montmorillonite are commercially available materials. High stiffness and brittleness and a limited thermal stability very close to the melting temperature make the processing conditions of P3HB difficult. The above-mentioned disadvantages of P3HB make it impossible to manufacture products with its participation on a commercial scale. The production of nanobiocomposites, the properties of which are analyzed in this work, improves its processing properties, extending the processing window by increasing the degradation temperature of these materials in relation to pure P3HB. This creates real possibilities for the production of these materials on an industrial scale and the production of utility products. The findings of this study are expected to contribute valuable insights into the design of sustainable biocomposites suitable for various applications, including packaging, agricultural films, and other environmentally friendly products.

## 2. Materials and Methods

### 2.1. Materials

P3HB was purchased from Biomer (Krailling, Germany) and Cloisite^®^30B from Southern Clay Products Inc. (Gonzales, LA, USA). Polyurethane based on 1,6-diisocyanate haxane (HDI) and polypropylene glycol with molecular weight 400 g·mole^−1^ (PPG400) was synthesized according to the procedure described in [41].

### 2.2. Methods

#### 2.2.1. Composite Production

**Figure 1:**
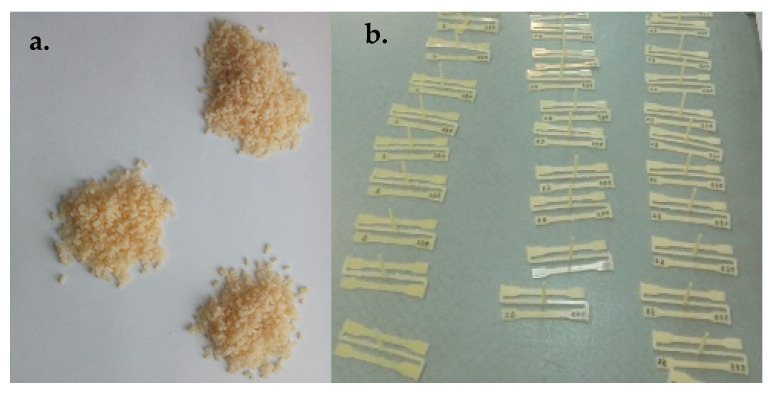
Physical pictures of the synthesized nanocomposites: (**a**) after extrusion and granulation and (**b**) after injection molding.

The production procedure for the nanobiocomposites was as described in [41]. The composition of the nanobiocomposites is given in Table 1. Nanocomposites were produced by mixing in a molten state with the use of a twin-screw extruder and granulator. The produced materials are shown in Figure 1.

#### 2.2.2. Mechanical Property Test Sample Preparation

Nanobiocomposite samples intended for mechanical testing were fabricated with a Dr BOY 55E injection molding machine as descried in [41]. The injection process was carried out according to the processing parameters given in Table 2.

The temperature settings of the plasticizing system, adjusted according to the PU content, are presented in Table 3.

Injection molding parameters were defined through an evaluation of pressure distributions inside the mold cavities, as recorded by the Priamus system (Schaffhausen, Switzerland) during each production cycle.

The trials revealed that material injection was unsuccessful when the mold was either cold or heated above 80 °C, as this led to the formation of significant shrinkage cavities in the molded parts. The material’s low thermal stability prevents it from remaining in the plasticizing cylinder for extended periods. Any interruptions in the injection process or prolonged plasticizing times causes thermal degradation of the sample.

#### 2.2.3. Monotonic Tensile Tests

The monotonic tensile measurements of the produced materials were performed using a biaxial, servo-hydraulic MTS 858 Mini Bionix testing system (Gliwice, Poland), featuring an axial load capacity of ±25 kN and a torsional load range of ±250 Nm. Longitudinal strain measurements were taken using an Instron 2620-601 axial extensometer (Norwood, MA, USA), with a gauge length of 75 mm and a measurement range of ±5 mm. To determine Poisson’s ratio (ν), a transverse extensometer (Instron 632.18F-20, Norwood, MA, USA) with a 10 mm gauge length and a ±2.5 mm range was additionally employed. Transverse strain was measured on the longer edge of the sample’s cross-section. The experimental procedure aimed to determine the fundamental mechanical properties of the nanobiocomposites, including Young’s modulus (Et); Poisson’s ratio (ν); yield strength (σy), where applicable; and ultimate tensile strength (σM) and corresponding strain (εM), as well as tensile strength at break (σB) and corresponding elongation (εB). All tests were conducted in accordance with the ISO 527-1 standard [42]. Additionally, for selected series, tensile tests were recorded using the Aramis 3D 4M digital image correlation (DIC) system (Oberkochen, Germany) to evaluate the evolution of stress and strain distributions across the gauge section of the specimens over time. For materials lacking a defined yield point according to ISO 527-1, the stress value σ₀.₀₁ corresponding to a strain of ε = 0.01 was used to facilitate comparison between sample series. Each test series included five samples, and the results were averaged. Standard deviation (s) was calculated for each measured parameter.

#### 2.2.4. Three-Point Bending Tests

The three-point bending tests of the prepared hybrid nanobiocomposites used an MTS Insight precision testing machine (Eden Prairie, MN, USA) with an electro-mechanical drive and a special three-point bending holder. The MTS Insight machine is characterized by a load range of up to 1 kN. The tests were carried out in accordance with the ISO 178 standard [43]. The loading speed was 5 mm/min. The aim of the research was to determine the modulus of elasticity *E*_f_; bending strength *σ*_fM_, and the corresponding strain *ε*_fM_; and stress at break *σ*_fB_, and the corresponding strain *ε*_fB_. Transverse strains were tested along the longer edge of the specimen cross-section. The experimental measurements focused on determining the fundamental mechanical properties of the resulted materials, including Young’s modulus (E_t_); Poisson’s ratio (ν); yield strength (σ_γ_), where applicable; and ultimate tensile strength (σ_m_), and its corresponding strain (*ε*_m_); as well as tensile strength at break (σ_β_), and the corresponding strain at break (*ε*_β_). All tests were conducted in accordance with the ISO 527-1 standard [42]. Additionally, for selected series, one sample from each was subjected to tensile testing while being recorded using the Aramis 3D 4M digital image correlation (DIC) system. This system was employed to capture the evolution of stress and strain distributions across the gauge section of the specimen over time. For materials without a defined yield point under the applicable standard, a reference stress value σ_0.01_ corresponding to a strain of *ε* = 0.01 was used to enable consistent comparison across different sample series.

#### 2.2.5. Biodegradability Test

The biodegradability of each composite based on a P3HB matrix was evaluated with the use of an OxiTop^®^ Control S6 device (WTW-Xylem, UA, San Diego, CA, USA), which works with a respirometric method to measure the oxygen demand required for the aerobic biodegradation of polymeric materials in soil. The oxygen consumption is expressed as biochemical oxygen demand (BOD): the amount of oxygen consumed per unit mass of the tested material.

The OxiTop^®^ Control S6 system consists of 510 mL glass bottles sealed with rubber stoppers and equipped with OxiTop-C measurement heads, enabling direct BOD monitoring. These measurement heads register pressure changes in the range of 500–1350 hPa with ±1% accuracy and operate effectively at temperatures between 5 °C and 50 °C. The setup also includes an OC 110 controller, which facilitates data transfer and communication between the measurement heads, the user, and the Achat OC software (v. 10.1, WTW-Xylem, UA, Baltimore, MD, USA) for analysis and interpretation of the biodegradation process.

The biodegradation study of the tested materials was conducted according to the ISO 17556:2012 standard [44]. Garden soil rich in humus was used as the degradation medium. The soil had the following characteristics: 5% moisture content (measured per ISO 11274 [45]), pH of 6 (ISO 10390 [46]), and a grain size of less than 2 mm. It was collected from Szczepanowo, located in the Kuyavian–Pomeranian Voivodeship (Poland).

For each test, 200 g of soil was placed into an OxiTop bottle, followed by the addition of 100 mL of distilled water. After thorough mixing, approximately 200 mg of the solid test material was introduced, and the exact sample mass was recorded. The bottle was then sealed with a rubber stopper containing two solid NaOH tablets, and the measurement head was attached. This procedure was repeated for all test and reference samples.

The bottles were placed in an incubator maintained at a constant temperature of 20 ± 0.2 °C and pre-incubated for 2 h. The BOD measurement program was then initiated using the OC 110 controller. The system remained in the incubator under constant temperature conditions for 28 days, with BOD values automatically recorded every 2–3 days.

The test series included:

- A blank sample (soil and water only),
- A positive control (starch: an easily biodegradable natural polymer),
- A negative control (polyethylene: a non-biodegradable polymer),
- And four tested samples: pure P3HB, and three composites containing 10% by mass polyurethane and increasing Cloisite^®^30B content (1%, 2%, and 3% by mass).

According to ISO 17556:2012, all test samples were prepared in similar size and shape, as the absolute degree of degradation is influenced by the geometry of the sample. The BOD for each bottle was calculated based on a formula that accounts for the oxygen demand of the test material, was corrected for the background BOD of the soil, and was normalized to the concentration of the test material within the soil.

(1) $${BOD}_{S}=\frac{{BOD}_{x}-{\;BOD}_{g}}{c}$$

where S is the number of measurement days; BOD_S_ is the biochemical oxygen demand of the analyzed sample within S days [mg/L]; BOD_x_ is the measured value for the tested sample [mg/L]; BOD_g_ is the measured value for the soil without sample [mg/L]; and c is the sample concentration in the tested system [mg/L].

The degree of biodegradation of the tested polymeric material was indicated in accordance with the following equation:

(2) $$D_{t}=\frac{{BOD}_{S}}{TOD}\cdot 100\%$$

where D_t_ is the degree of material biodegradation [%] and TOD is the theoretical oxygen demand [mg/L]. The TOD for each system was determined according to the formula included in the standard. It was considered that, under aerobic conditions, the biodegradation process would cause that carbon present in the tested material is transformed into CO_2_, hydrogen into water, phosphorus into phosphorus oxide (P_4_O_10_), sulfur into sulfur trioxide, nitrogen into ammonia, and chlorine into hydrogen chloride. For a compound with a known molecular formula containing the elements C, H, Cl, N, S, P, Na, and O, the TOD value was calculated using the following equation:

(3) $$TOD=\frac{16[2c+0.5\left(h-cl-3n\right)+3s+2.5p+0.5k-o]}{M_{r}}$$

where c, h, p, s, n, cl, k, and o are the quantities of each of the elements in a particle of tested sample [-] and M_r_ is the weight of the tested sample [g].

#### 2.2.6. Infrared Spectroscopy Measurements

The confirmation of specific groups in the composites was analyzed using Fourier transformation infrared spectroscopy (FTIR). FTIR spectra were recorded using a Thermo Fisher Scientific Nicolet iS20 spectrophotometer (Waltham, MA, USA) in the range from 400 to 4000 cm^−1^. A solid sample placed on a measuring socket was used for the analysis.

#### 2.2.7. Thermal Analysis

The thermal properties of P3HB and the fabricated polymer nanobiocomposites were analyzed by thermogravimetric method (TGA). The measurements were carried out with the use of a Melter Toledo TGA/SDTA 851e analyzer (Columbus, OH, USA). The samples were heated at a rate of 5 °C per minute within a temperature range of 25 °C to 600 °C under a nitrogen atmosphere. The following thermal degradation parameters were evaluated: onset degradation temperature (Ton); temperature at which 50% of the mass is lost (T_50%_); temperatures corresponding to 10% and 5% mass loss (T_10%_, T_5%_); temperature of the peak degradation rate (T_max_); and residual mass remaining at 600 °C.

#### 2.2.8. DSC Analysis

The heat parameters of the tested samples were measured with the use of a Differential Scanning Calorimeter (DSC822e, Mettler Toledo, Columbus, OH, USA). The relationship of the heat flow in the function of the temperature was investigated. All measurements were performed under a nitrogen atmosphere at a 50 mL·min^−1^ flow rate. The temperature range spanned from −90 °C to 220 °C. The accuracy of the specific heat measurements was approximately 3%. Both cooling and heating rates were set at 10 °C·min^−1^. A second heating cycle was used to assess their properties.

#### 2.2.9. Elemental Analysis

Elemental analysis in the mode (CHN) of P3HB and its composites with PU and Cloisite 30B was performed using a Vario EL III C, H, N analyzer from the company Elementar (Langenselbold, Germany).

## 3. Results and Discussion

**Figure 2:**
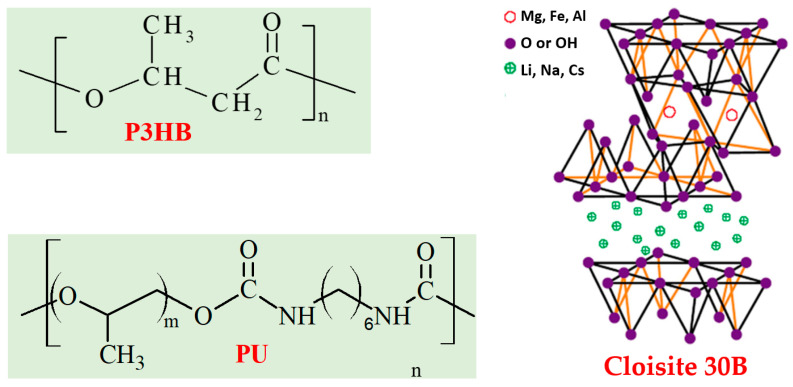
Structure of the components the hybrid nanobiocomposites.

In this work, mechanical, thermal, and biodegradability tests were conducted on P3HB-based hybrid polymer nanobiocomposites, with 10% by mass of thermoplastic polyurethane. PU was synthesized during reaction of polypropylene glycol and HDI (Figure 2). The organically modified montmorillonite, commercially known as Cloisite^®^30B, was used in amounts of 1, 2, and 3% by mass (Table 1) as a nanoadditive. The polymer hybrid nanocomposites were prepared by mixing in a molten state with the use of a twin-screw extruder. P3HB and its composition with PU were processed in the same way.

### 3.1. Mechanical Properties of the Hybrid Nanobiocomposites

**Figure 3:**
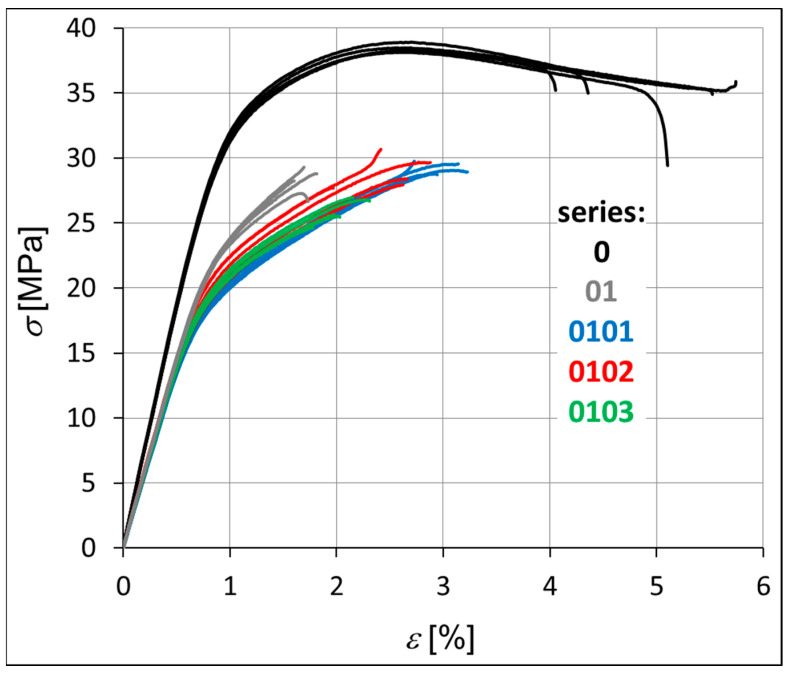
Monotonic tensile curves of the investigated materials, i.e., P3HB and materials of series 01, 0101, 0102, and 0103.

**Figure 4:**
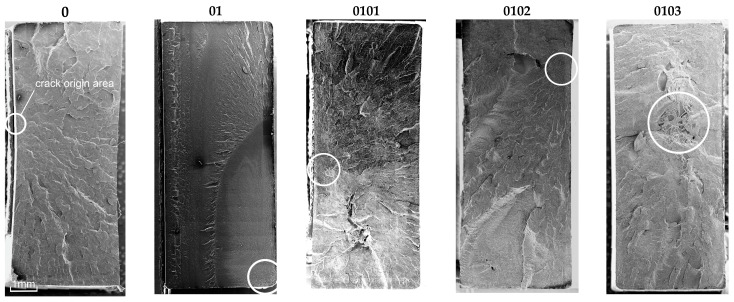
Macro views (SEM) of the fracture surfaces obtained in monotonic tensile tests (gold sputtered surfaces) for native P3HB and materials of series 01, 0101, 0102, and 0103, with crack origin areas marked.

**Figure 5:**
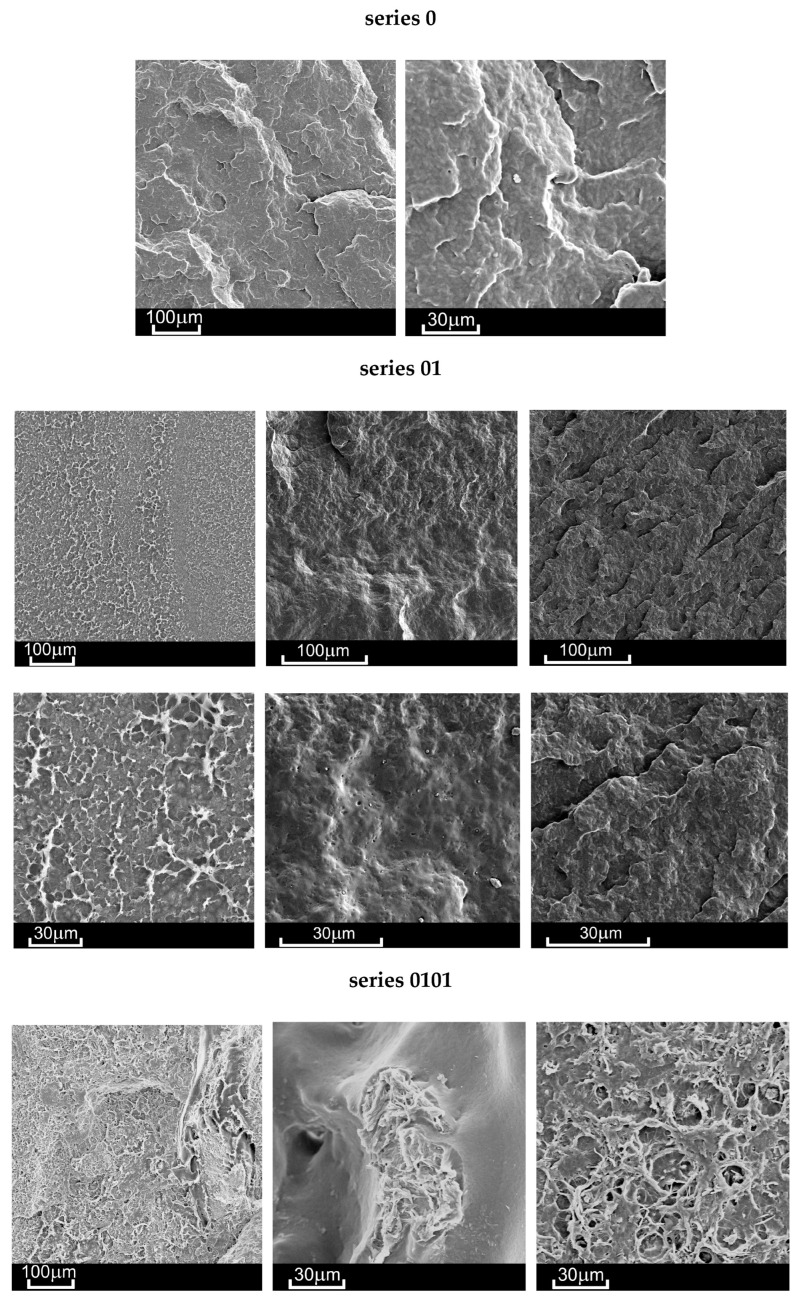

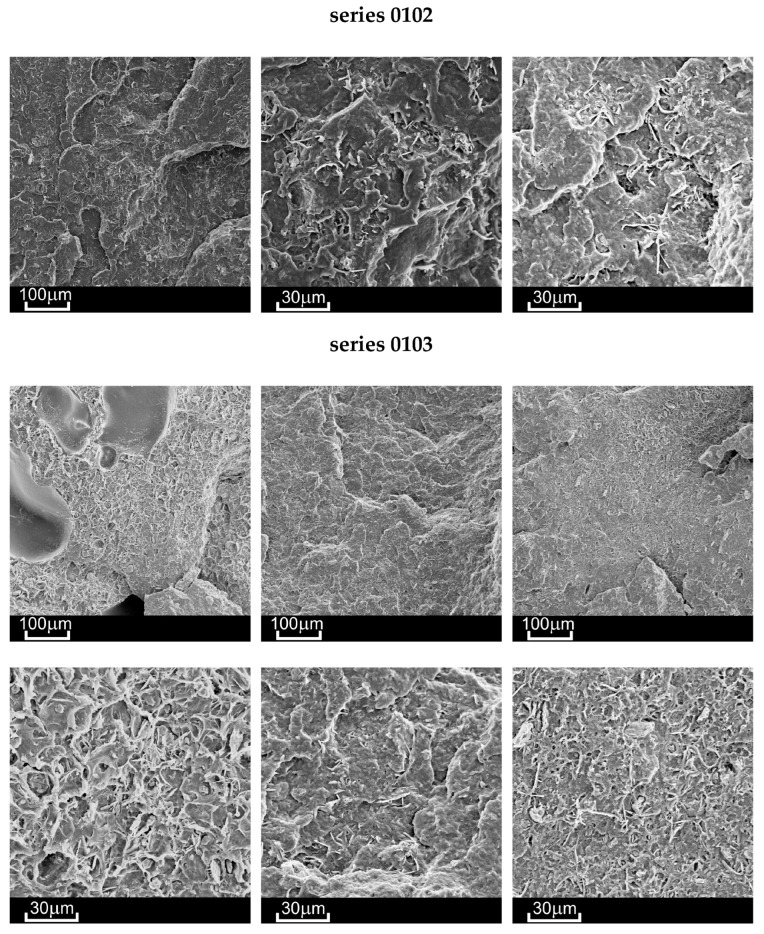
SEM views of fracture surfaces obtained in monotonic tensile tests of the investigated materials (gold sputtered surfaces) for native P3HB and materials of series 01, 0101, 0102 and 0103.

The mechanical strength of each hybrid nanobiocomposite was tested in monotonic tensile tests. Figure 3 shows the monotonic tensile curves obtained for all series, i.e., P3HB–0, P3HB–PU composition–01, and nanobiocomposites 0101, 0102, and 0103. Table 4 presents the average values of strength parameters obtained for five specimens. The value of the standard deviation (*s*) is shown in brackets next to the parameter value. It is worth noting the overall repeatability of results within each series, with the exception of series 0102, which consistently shows the greatest variability (Figure 3, Table 4). Within each series, the most significant discrepancies are observed in the strain at break (*ε*_B_). This phenomenon is not exclusive to polymeric materials but is also commonly reported for metals and their alloys. The strain at break is often highly influenced by local imperfections in the sample geometry or by microstructural irregularities within the material itself. This is clearly visible in Figure 4 and Figure 5 in the form of pores created during the process of injecting the material into the mold. The base material has the smallest number of voids. The material of series 01, 0101, 0102 and 0103 is less stiff than the P3HB.

The results indicate that the unmodified P3HB (series 0) can be classified as a rigid material exhibiting a distinct yield point (σ_y_). In this case, the yield strength equals the ultimate tensile strength (*σ*_y_ = *σ*_M_). In contrast, the materials from the modified series do not exhibit a clearly defined yield point according to the relevant standards, and thus for these materials, the strength at break is considered equivalent to the maximum stress (*σ*_B_ = *σ*_M_).

The stiffness of the tested samples in series 01, 0101, 0102, and 0103 is significantly lower compared to that of the pure P3HB, with a reduction of approximately 24% (Table 4). Consequently, these series also demonstrate an increase in Poisson’s ratio (ν) relative to the base material. Both Young’s modulus (E_t_) and Poisson’s ratio remain relatively consistent across series 0101, 0102, and 0103. The main differences within these series are observed in the stress and strain at break (*σ*_B_, *ε*_B_), with series 0103 showing a noticeable decrease in both parameters. In contrast, series 01, 0101, and 0102 exhibit higher values, particularly for elongation at break (*ε*_B_). The addition of polyurethane to the base polyester (series 01) significantly reduces its ductility. However, further modification by incorporating montmorillonite clay leads to an increase in ductility, with the most pronounced effect observed for the lowest clay content (series 0101), as illustrated in Figure 3.

The macro fracture view shows that the crack initiation process can take place on the outer surface of the sample or at the border of a defect (void, pore) inside the material (Figure 4 and Figure 5). In the photos in Figure 4, the crack initiation areas are marked with white circles. The crack propagation process is very visible in the case of the native P3HB (series 0). In the case of the 01 series, the boundary between the propagation area and the rapid, final failure area is clearly visible. However, the increase in the addition of organic clay causes the failure of the material to occur more and more rapidly, i.e., with an increasingly shorter stage of crack propagation. It is worth noting that the 01 series material has the smallest number of voids. SEM images prove that the material of series 0101, 0102 and 0103, apart from large-sized voids, also has much smaller pores. The addition of organic clay causes characteristic fibers to appear in the structure of the material. Their distribution in different areas of the fracture surface is irregular. However, an increasing trend in the number of these fibers can be observed as the percentage of clay in the base material increases.

As can be seen in Figure 4 the fracture surface of the polyester matrix was slightly wavy and glassy, which indicates a regular crack propagation path. PU added to the matrix reacts with P3HB and causes a decrease in the elongation ε during the application of force (Figure 3), which may be related to the reduced movement of the chains, and thus the disruption of the crystal structure of P3HB itself. In the micrographs of the two-component composition (Figure 5), we can therefore observe the separation of the glassy areas of P3HB by the PU bound to it, and the structure of the composite is the same throughout the fracture of the sample. Hence, the force during the tensile and rupture tests are the same for sample “0” and the binary composition 01. The structures of the hybrid samples, i.e., containing PU and various amounts of Cloisite, are already different from those previously discussed. The introduced MMT constitutes an internal skeleton in the composites in which the areas formed from the connected PU chains with P3HB are embedded. However, the more Cloisite 30B there was in the sample, the more unevenly distributed a skeleton it created in the entire volume of the sample. In samples 0102 and 0103 (Figure 5) we can see clusters of nanoclay, which indicates the heterogeneity of the structure of these nanocomposites. In addition, pores were identified in all nanocomposites. Their number is greater when there is less MMT in the sample. The identified pores, occurring in a negligible amount, can act as stress concentrators, affecting the enhancement of the mechanical properties of the nanocomposites. The discussed stiffening by the nanoclay skeleton translates into a reduction in the deformation of the samples during bending and tearing tests. According to previous analyses, it seems that the sample containing the smallest, i.e., 1% by weight Cloisite, exhibits the best mechanical properties. The evenly distributed skeleton created from organophilic MMT maintains the interacting matrix and polyurethane polymeric modifier. This amount of nanoclay seems to be optimal.

**Figure 6:**
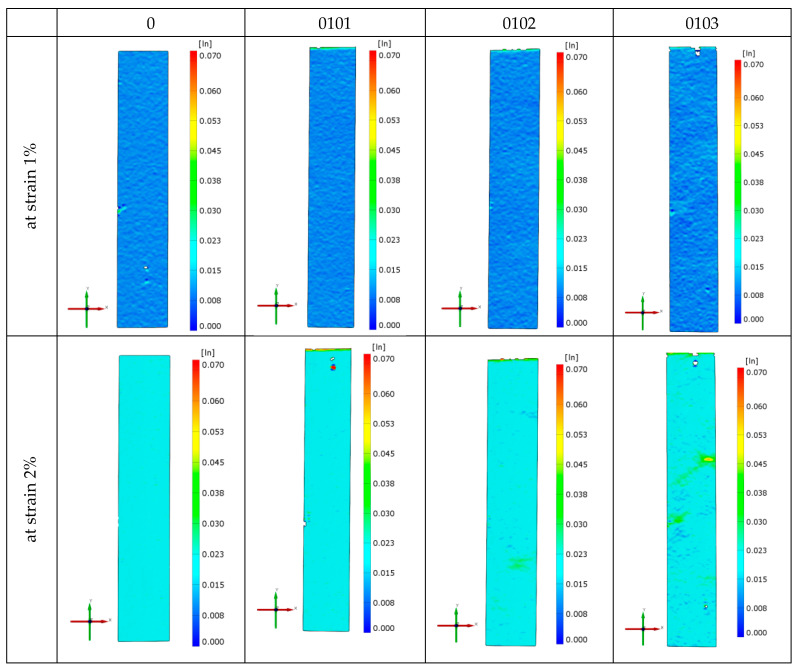

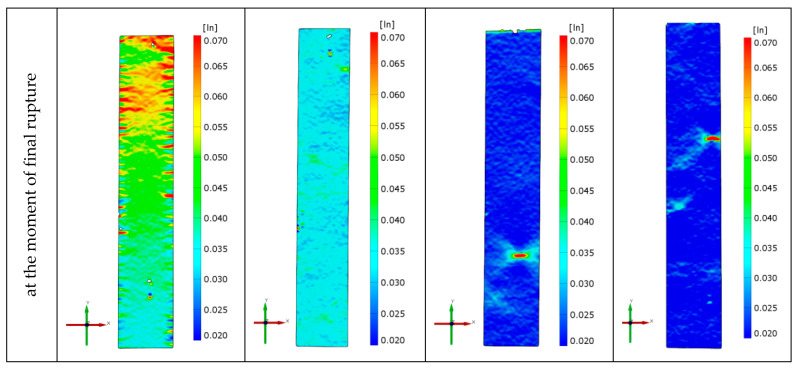
Strain fields obtained during monotonic tensile tests for the native P3HB and materials of series 01, 0101, 0102, and 0103.

Figure 6 shows the strain fields of exemplary specimens of the investigated material obtained in monotonic tensile tests using the Aramis 3D 4M vision system. The distributions of the strain field in the entire measuring part of the sample are presented here for the relative strain ε of 1% and 2%, as well as just before the sample breaks. In the case of *ε* = 1%, the distribution of the strain field in the entire area of the specimen gauge length is uniform. For *ε* = 2%, it can be seen that the heterogeneity of the mentioned distributions increases with the increase in the percentage of organic clay in the P3HB–PU matrix. This heterogeneity appears in places where the pores are located close to the specimen wall. In such cross-sections, the final rupture of the specimen usually occurs. In the case of the native P3HB (series 0), numerous small micro-cracks are visible on the specimen surface.

**Figure 7:**
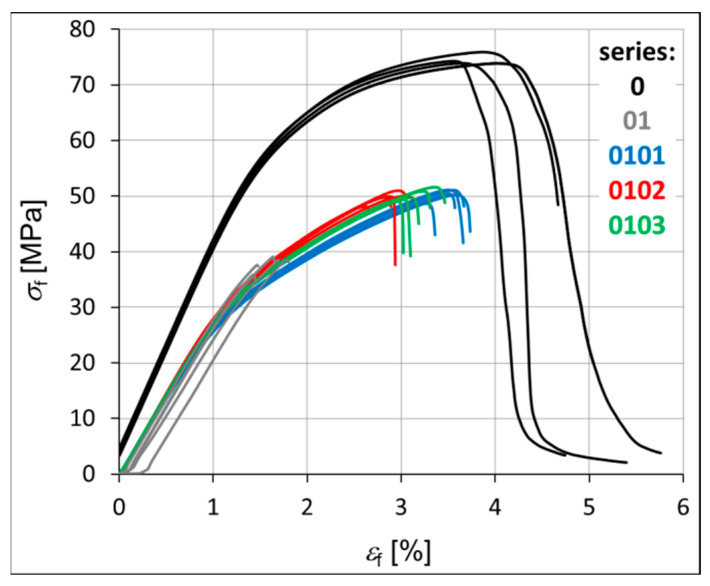
Monotonic bending curves for the investigated materials, i.e., for the native P3HB and materials of series 01, 0101, 0102, and 0103.

The prepared nanobiocomposites were also tested in three-point bending. The aim of the test was to determine modulus of elasticity *E*_f_; bending strength *σ*_fM_, and the corresponding strain *ε*_fM_; and stress at failure *σ*_fB_, and the corresponding strain *ε*_fB_. The test results of the three-point bending are summarized in Table 5 and in Figure 7 to the relevant standard. Table 3 shows the average values of strength parameters obtained for five specimens. The value of the standard deviation (*s*) is shown in brackets next to the parameter value. The native P3HB belongs to the group of materials showing the maximum stress *σ*_f_ during the bending test and cracking before reaching conventional deflection *s*_C_. In turn, the standard classifies materials from series 01, 0101, 0102, and 0103 as materials rupturing before plastic strain and then *σ*_fM_ = *σ*_fB_. The values of the longitudinal modulus of elasticity *E* determined in monotonic tensile tests and three-point bending tests coincide (Table 4 and Table 5). The stress at break *σ*_fB_ for material series 01, 0101, 0102, and 0103 remain at an almost identical level of 50 MPa, while for the base material (series 0) it is much higher. The strain at break *ε*_fB_ is the largest for the base material. It decreases for material series 0101, 0102, and 0103 with increasing percentage of organic clay. We can observe a similar situation in tensile tests.

The impact strength tests results given in Table 6 show that the lowest impact strength was that of native P3HB (series 0), for which a value of 7.94 J/cm^2^ was determined. Introduction of PU in amount of 10% by mass resulted in an increase of impact strength to the value of 9.23 J/cm^2^. A beneficial effect of the addition of organic clay was observed for the materials of series 0101, 0102, and 0103, which were characterized by a higher impact strength than the native P3HB. The highest values were noted for the 0101 series of specimens in which 1% Cloisite^®^30B was used, for this series an increase in impact strength of almost 113% was observed. On the other hand, increasing the nanoparticle content by 2 and 3% reduced the impact strength but it was still higher, at 96% and 55%, respectively, than native P3HB. Data from the literature indicate a positive effect of nanoparticles in improving the impact properties of polymer composites [47,48,49]. The increase in impact strength of polymer composites with nanoparticles may be due to better stress dispersion, improved interfacial interactions, and increased fracture toughness, leading to more efficient dissipation of impact energy. The paper [47] analyzed the effect of clay and silica nanoparticles on the impact resistance of the composites. The results showed that all samples with nanoparticles exhibited increased impact strength compared to samples without modification. The authors indicated that nanoparticles result in a more homogeneous structure, which translates into better fracture toughness. In a separate study [49], glass-fiber-reinforced composites incorporating carbon nanotubes exhibited a 17% increase in impact strength. The incorporation of hybrid nanoparticles led to progressive improvements in tensile, flexural, and impact properties as their concentration increased. However, upon exceeding the optimal filler loading threshold, a deterioration in mechanical performance was observed due to potential nanoparticle agglomeration effects [50].

The selected mechanical properties, mainly tensile strength, of the resulted P3HB-based nanobiocomposites and its binary composition with PU were compared to other compositions and composites with polyester (P3HB) matrix as it is given in Table 7. The addition of another polymer to P3HB, e.g., PCL [12], PLA [51], or chitosan [13], causes a decrease in the tensile strength of the obtained binary composition. Similarly, the introduction of a nanofiller to the P3HB matrix, e.g., CNCs, also decreases the tensile strength of the composite [52]. The production of nanocomposites using P3HB and sepiolite nanoclay results in a slight increase in strength, as higher a concentration of sepiolite was added [8]. The strain at break also decreases after the addition of a polymer modifier or nanofiller [8,13,51,52,53]. An exception is the composition of P3HB with PCL, which is characterized by a significant increase in elongation by more than two times [12]. The bending strength of this composition decreases. The introduction of sepiolite nanoclay into P3HB causes a different effect than in the case of the nanocomposites described in this article, namely a decrease in impact strength was observed [8].

### 3.2. Biodegradability of the Resulted Hybrid Polymer Nanobiocomposites

The biodegradability test was performed on P3HB, its PU blend, and three samples of hybrid nanobiocomposites, with elemental compositions as given in Table 8, and the results are given in Table 9.

Based on the 28-day respirometric biochemical oxygen demand (BOD) test, native P3HB exhibited a biodegradability degree of 63.21% (Table 9), indicating a high level of biodegradability. This result confirms that P3HB meets the criteria for classification as a biodegradable polymer. The polymer composition consisted of 90% by mass of polyester and 10% by mass of thermoplastic polyurethane based on aliphatic isocyanate HDI (01). The remaining three additionally contained an increasing amount of Cloisite 30B nanofiller from 1 wt.% to 3 wt.% (0101, 0102, and 0103). The biodegradability test of sample 01 showed complete decomposition of the composite during the test (the degree of biodegradability was 100%). Complete biodegradability of the sample was also confirmed by screening the biodegradation environment after the test, which did not reveal any residues of the polymer composite. This proves that the composition used is completely biodegradable, and the presence of linear structures in the polyurethane does not inhibit biodegradation, but on the contrary promotes its occurrence. The use of a polyurethane modifier as a hydrophilic polymer increases the water absorption into the polymer mass and accelerates its hydrolysis [54].

Ikejima et al. observed a similar influence of PVA presence in the blends of P3HB/PVA for the BOD value. Blended P3HB/PVA (90/10) has higher a BOD value (61%) compared to pure P3HB (55%), whereas pure PVA has a lower BOD value (25%) [9]. In turn, Ikejima and Inoue tested the biodegradability of blended P3HB/chitosan and P3HB/chitin [55]. They measured BOD values of 70, 66, and 63% for P3HB, P3HB/chitin (75/25), and P3HB/chitosan (75/25), respectively. Thus, the presence of chitin and chitosan caused lower degradability.

Modification of composition 01 with a nanofiller had a significant effect on the biodegradability value. Each time, a decrease in this parameter was noted with an increase in the amount of nanofiller, to 82.35% for 1 wt.% of nanofiller (0101), to 78.04% for 2% by mass of nanofiller (0102), and 55.66% for 3 wt.% of nanofiller (0103) (Table 9). The obtained results confirmed that the presence of nanofiller inhibits the biodegradation process. Already 3% by mass reduces the efficiency of this process by almost half. It should be emphasized that nanobiocomposites 0101 and 0102 have higher biodegradability than native P3HB.

**Figure 8:**
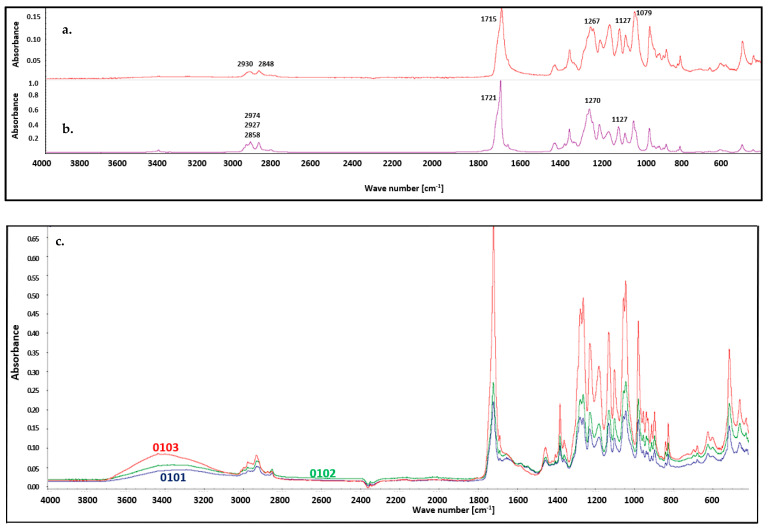
FT IR spectra of P3HB: (**a**) after biodegradation, (**b**) before biodegradation, and (**c**) hybrid nanobiocomposites with 1, 2 and 3% by mass of Cloisite 30B, series 0101, 0102, and 0103, respectively, conducted after biodegradability the tests.

FT IR analysis of the materials was performed after the biodegradability tests were conducted. In Figure 8 shows native P3HB’s spectrum before biodegradation. The presence of characteristic absorption bands of P3HB confirmed its structure and allow for the structural identification of the matrix in the blend. A distinct ester carbonyl absorption band, corresponding to the C=O stretching vibrations, was observed at 1718 cm^−1^. Additionally, the asymmetric and symmetric C–O stretching vibrations of the ester group were observed at 1270 cm^−1^ and 1127 cm^−1^, respectively. The spectrum also exhibited bands corresponding to the asymmetric and symmetric C–H stretching vibrations of methyl and methylene groups at 2974, 2927, and 2858 cm^−1^ [56]. Basically, the FTIR spectra of the residues after biodegradation do not show any major changes (Figure 8), with the exception of a smaller intensity for all bands, which resulted from the biodegradation process. Nevertheless, a shift of the C=O stretching vibration band to a lower wavenumber, from 1721 cm^−1^ to 1715 cm^−1^, was observed, which is attributed to the formation of hydrogen bonds with water [57]. A band of different intensity was noted at 3400 cm^−1^ in each spectrum after biodegradation, which was caused by the adsorption of water from the biodegradation environment [58,59]. Moreover, the bands corresponding to the asymmetric and symmetric C–H stretching vibrations of methyl and methylene groups in the 2800–2950 cm^−1^ region became less sharp and more broadened.

The FT IR spectra of samples with nanofillers show similar bands. One difference is the appearance of a characteristic low-intensity band around 1596 cm^−1^, attributed to the deformation vibrations of the N–H bonds in the polyurethane groups. Another difference is the slightly higher intensity of all bands compared to the spectra of native P3HB, which is related to the reduced extent of biodegradation. Additionally, the intensity of the band at 3400 cm^−1^ increases with increasing Cloisite^®^30B content in the nanocomposites. This increase results from stronger interactions with water and, on the other hand, a lower tendency toward biodegradation [60].

### 3.3. Thermal Properties of Polymer Hybrid Nanobiocomposites

**Figure 9:**
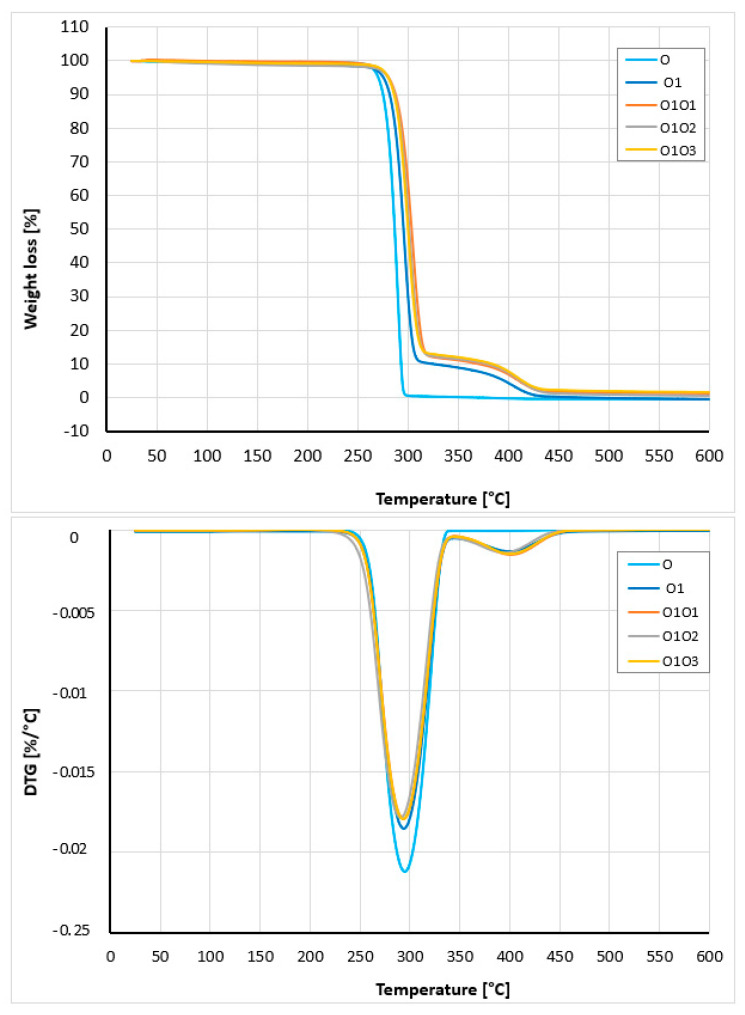
TG and DTG curves of P3HB (0), the blend P3HB–PU (01), and nanobiocomposites containing 1, 2, and 3% by mass Cloisite 30B (0101, 0102, and 0103), obtained at a heating rate of 5 °C/min under a nitrogen atmosphere.

The thermal stability of the produced hybrid nanobiocomposites was evaluated by thermogravimetric analysis (TGA) and compared to that of the P3HB–PU polymer blend and native P3HB. The corresponding TG and DTG curves are shown in Figure 9. The interpretation of the curves is included in Table 10. Comparing the thermal stability of the hybrid nanobiocomposites, the highest onset decomposition temperature (263 °C) was observed for sample 0101, indicating superior thermal stability for this composition. The remaining nanobiocomposites showed a lower decomposition onset temperature, with 0103 being 253.7 °C. For comparison, the decomposition onset temperature of P3HB (0) was 232.8 °C, and its composition with PU was 255.5 °C. The presence of 1% by mass of organic nanoclay noticeably enhances the thermal stability of the hybrid nanobiocomposites. The more noticeable effect of Cloisite 30B is at 5% mass loss, then the temperatures rise to 275.8, 285.1, 280.8, and 286.3 °C for composition 01 and nanobiocomposites 0101, 0102, and 0103, respectively (Table 10). The decomposition of the P3HB–PU composition (01) occurs in two stages. First, the main stage of degradation occurs between 230 to 330 °C, followed by a secondary degradation stage in the range of 330 °C to 440 °C. In turn, the decomposition of P3HB is single-stage in the range of 230 to 330 °C. This indicates that the decomposition of PU in composition (01) and nanobiocomposites (0101, 0102, and 0103) occurs in the second stage. The presence of residues at a temperature of 600 °C is small, approx. 1.4–1.9% by mass for bionanocomposites, and is related to the presence of inorganic aluminosilicate. Kotal and Bhowmick observed a similar influence of Cloisite on the thermal stability of nanocomposites based on poly(styrene-co-butyl acrylate) [61]. Nanocomposites based on PMMA and organomodified montmorillonite are also characterized by higher thermal stability then neat PMMA [62].

The qualitative thermal analysis using differential scanning calorimetry (DSC) of materials P3HB (series 0), composition P3HB–PU (series 01), and their nanobiocomposites (series 0101, 0102, and 0103) was conducted based on the analysis of heat flow rate as a function of temperature; the glass transition and melting regions were identified, confirming the semicrystalline nature of P3HB and its nanobiocomposites. The phase transition temperatures are summarized in Table 10. The study on the impact of Cloisite^®^30B on the thermal properties of poly(3-hydroxybutyrate) (P3HB) and its nanobiocomposites revealed that the addition of polyurethane (PU) to P3HB led to a decrease in the glass transition temperature (Tg). Further incorporation of PU and Cloisite^®^30B resulted in additional reductions in Tg, indicating a plasticizing effect of both components. The most pronounced change was observed in sample series 0103, where Tg decreased by 25.3 °C compared to pure P3HB (series 0). A similar trend was reported for nanocomposites based on P3HB with other PU and Cloisite^®^30B [63]. Conversely, Dayma and Satapathy [64] observed an increase in Tg for polyamide-6/LDPE-g-MA/Cloisite^®^30B nanocomposites.

The incorporation of polyurethane also reduced the melting point of P3HB from 157.5 °C to 128.5 °C, a decrease of approximately 29 °C. However, the addition of both PU and Cloisite^®^30B (series 0101) increased the melting point to 136.3 °C, which remains 20.8 °C lower than that of pure P3HB. The increase in nanoclay concentration in the composites caused the Tm to decrease by a further 4.5 °C (series 0103), but not below the Tm of composition (01). Loyens et al. [65] also observed a reducing melting point for nanocomposites of poly(ethylene oxide)/Cloisite30B after adding nanoclay and with the increase of its concentration. Similarly, Botana et al. described nanocomposites of P3HB/Cloisite30B characterized by a lower melting point than P3HB [51].

## 4. Conclusions

The addition of organomodified montmorillonite to a polymer composite P3HB with PU significantly reduced the stiffness of the material, which led to a decrease in tensile strength. However, an increase in ductility was observed, especially with lower amounts of montmorillonite. Increasing the amount of montmorillonite improved the material flexibility but also accelerated crack propagation, which could reduce the overall durability of the material.

SEM observations showed that samples with higher montmorillonite content had smaller pores and characteristic fibers, which may influence the fracture behavior of the material. Increasing the montmorillonite content in the material led to a more irregular distribution of these fibers, affecting the material’s behavior during the tensile and bending tests.

Adding montmorillonite to the P3HB–PU matrix enhanced thermal stability by raising the onset temperature of decomposition, especially in samples containing 1% by mass of modified montmorillonite. A higher montmorillonite content (up to 3% by mass) negatively impacted and decreased the thermal stability.

P3HB exhibits high biodegradability, reaching 63.21%, confirming its biopolymer nature. The addition of PU and montmorillonite improved biodegradability, with the highest biodegradation observed for the PU composition (100%). However, as the montmorillonite content increased (up to 3% by mass), the biodegradation efficiency gradually decreased, with 1 and 2% by mass modified montmorillonite bionanocomposites showing better biodegradation than native P3HB.

Hybrid nanobiocomposites based on P3HB–PU with organically modified montmorillonite show promising mechanical, thermal, and biodegradation properties. However, higher modified montmorillonite concentrations negatively affect biodegradation and material durability. The best overall properties were obtained for the material with 1% by mass of the organomodified montmorillonite.

## Figures and Tables

**Table 1:** Composition of the hybrid nanobiocomposites.

Share of P3HB [% by Mass]	Share of PU [% by Mass]	Share of Cloisite^®^30B [% by Mass]	Sample Designation
90	10	0	01
89	10	1	0101
88	10	2	0102
87	10	3	0103

**Table 2:** The injection molding settings for P3HB-based biocomposite samples containing 10 wt.% PU and 0, 1, 2, or 3 wt.% Cloisite^®^30B, identified as 01, 0101, 0102, and 0103, respectively, for mechanical testing.

Parameter	Value
Filling Rate [cm^3^/s]	40
Filling pressure [bar]	550–600
Packing pressure (profile) [bar]	300–350
Packing time (profile) [s]	23
Mold temperature [°C]	40
Plasticization pressure [bar]	150

**Table 3:** The thermal profile programmed into the injection molding machine for processing P3HB nanobiocomposites containing 10% by mass linear PU and 0, 1, 2 or 3% by mass of the nanoclay Cloisite^®^30B, respectively marked as 01, 0101, 0102, 0103.

Series	T_1_ [°C]	T_2_ [°C]	T_3_ [°C]	T_4_ [°C]	T_Nozzle_ [°C]
0	170	174	177	179	180
01	170	173	175	178	178
0101	168	172	176	177	177
0102	168	172	174	177	177
0103	168	172	174	177	177

**Table 4:** Measured values of key strength parameters for the examined materials, P3HB and materials of series 01, 0101, 0102, and 0103, together with the standard deviation *s* (in brackets).

Series	*E*_t_ (*s*) [GPa]	*ν* (*s*) [-]	*σ*_y_ (*s*) [MPa]	*σ*_0.01_ (*s*) [MPa]	*ε*_y_ (*s*) [%]	*σ*_B_ (*s*) [MPa]	*ε*_B_ (*s*) [%]
0	3.73 (0.041)	0.38 (0.006)	38.43 (0.27)	–	2.65 (0.085)	35.66 (0.76)	4.82 (0.61)
01	2.91 (0.023)	0.352 (0.017)	–	23.66 (0.16)	–	28.14 (0.97)	1.67 (0.11)
0101	2.76 (0.018)	0.402 (0.009)	–	20.2 (0.2)	–	29.13 (0.45)	2.97 (0.19)
0102	2.86 (0.076)	0.396 (0.007)	–	21.69 (0.73)	–	28.87 (1.12)	2.51 (0.31)
0103	2.82 (0.031)	0.397 (0.007)	–	21.03 (0.26)	–	25.85 (0.87)	2.05 (0.18)

**Table 5:** Values of basic parameters obtained in the three-point bending test for investigated materials, i.e., for the unmodified P3HB (0), P3HB–PU composition (01), and their nanocomposites of series 0101, 0102, and 0103 together with standard deviation *s* (in brackets).

Series	*E*_f_ (*s*) [GPa]	*σ*_fM_ (*s*) [MPa]	*ε*_fM_ (*s*) [%]	*σ*_fB_ (*s*) [MPa]	*ε*_fB_ (*s*) [%]
0	3.75 (0.027)	74.37 (0.81)	3.75 (0.17)	63.45 (1.63)	4.25 (0.26)
01	2.86 (0.162)	–	–	37.94 (1.13)	1.60 (0.14)
0101	2.74 (0.059)	–	–	50.31 (0.89)	3.48 (0.11)
0102	2.87 (0.059)	–	–	49.77 (0.93)	2.86 (0.14)
0103	2.84 (0.018)	–	–	50.15 (0.94)	3.13 (0.14)

**Table 6:** Average unnotched Charpy impact strength values for P3HB (0), the P3HB–PU com-position (series 01), and their nanobiocomposites with 1, 2, and 3% by mass of Cloi-site^®^30B (series 0101, 0102, and 0103).

Series	Impact Strength [J/cm^2^]
0	7.94 ± 0.3
01	9.23 ± 0.6
0101	16.88 ± 0.8
0102	15.53 ± 0.4
0103	12.26 ± 0.5

**Table 7:** Comparison of the selected mechanical properties of polyester-based materials.

Material	Tensile Strength [MPa]	Elongation at Break [%]	Flexural Strength [MPa]	Impact Strength [J/cm^2^]
P3HB	36	4.8	63.5	7.9
P3HB/PU (90/10)	28	1.7	37.9	9.2
P3HB/Cloisite30B (95/5) [51]	27	2.5	-	-
P3HB/PCL (75/25) [12]	23	12.5	37.5	-
P3HB/Chitosan (90/10) [13]	13	3	-	-
P3HB/PLA (75/25) [52]	24	2.9	-	
P3HB/CNCs (98/2) [8]	30	1.5	-	
P3HB/CNCs (96/4) [8]	30	1.5	-	
P3HB [53]	38.46	5.8	-	4.1
P3HB/sepiolite (99/1) [53]	39.67	5.3	-	4.4
P3HB/sepiolite (98/2) [53]	40.06	4.0	-	3.0
P3HB/sepiolite (97/3) [53]	40.25	3.5	-	2.7

**Table 8:** The elemental composition of P3HB, the P3HB–PU blend, and the hybrid nanobiocomposites was determined both theoretically—based on the chemical structure of the components—and experimentally via elemental (CHN) analysis.

Series	C		H		O		Si		N	
	Calcd	Determined	Calcd	Determined	Calcd	Determined	Calcd	Determined	Calcd	Determined
0	0.5581	0.5595	0.0698	0.0694	0.3724	0.3711	0	-	0	-
01	0.5616	0.5605	0.0724	0.07268	0.3623	0.3575	0	-	0.0094	0.0093
0101	0.5560	0.5550	0.0717	0.07195	0.3639	0.3642	0.0047	-	0.0094	0.0089
0102	0.5504	0.5504	0.0710	0.07101	0.3655	0.3702	0.0093	-	0.0095	0.0094
0103	0.5449	0.5466	0.0703	0.07098	0.3671	0.3725	0.0140	-	0.0096	0.0099

**Table 9:** Summary of BOD measurements, theoretical oxygen demand, and biodegradability degree for the tested samples.

Series	Sample Mass [g]	TOD [mg/L]	BOD Measured [mg/L]	Sample BOD [mg/L]	Dt—Degree of Biodegradability [%]
0	0.25	49.84	60.00	31.50	63.21
01	0.22	57.54	86.30	57.80	100.00
0101	0.25	50.39	70.00	41.50	82.35
0102	0.19	65.99	80.00	51.50	78.04
0103	0.16	77.98	71.90	43.40	55.66

TOD—theoretical oxy17, lines 520-gen demand [mg/L], BOD measured—biochemical oxygen demand of the analyzed material with soil, sample BOD—measurement results for the material alone, and Dt—degree of material biodegradability.

**Table 10:** Interpretation of TG and DTG curves of P3HB (0), its biocomposition with PU (01), and their nanobiocomposites with 1, 2, and 3% by mass of Cloisite^®^30B (0101, 0102, and 0103) obtained at a heating rate of 5 °C/min under a nitrogen atmosphere. Values of glass transition temperatures and melting points of the tested materials were determined from DSC measurements.

Sample	T_on_ (°C)	T_5%_ (°C)	T_10%_ (°C)	T_50%_ (°C)	T_max_ (°C)	Residue at 600 °C (% by Mass)	Tg (°C)	Tm_(onset)_ (°C)
0	232.8	270.0	274.7	287.2	295.7	0.10	5.5	157.5
01	255.5	275.8	281.7	295.0	294.5	0.23	−18.4	128.5
0101	263.0	285.1	291.5	307.3	293.5	1.67	−19.1	136.3
0102	260.5	280.5	287.5	301.8	294.5	1.40	−20.2	133.2
0103	253.7	286.3	292.2	306.2	294.3	1.90	−20.8	131.8

## Data Availability

The original contributions presented in this study are included in the article. Further inquiries can be directed to the corresponding author.

## References

[1] Dahman Y., Ugwu C.U. (2014). Production of green biodegradable plastics of poly(3-hydroxybutyrate) from renewable resources of agricultural residues. Bioprocess Biosyst. Eng..

[2] García A., Aguirre C., Pérez A., Bahamonde S.S., Urtuvia V., Díaz-Barrera A., Peña C. (2024). Recent Trends in the Production and Recovery of Bioplastics Using Polyhydroxyalkanoates Copolymers. Microorganisms.

[3] de Sousa Junior R.R., Cezario F.E.M., Antonino L.D., dos Santos D.J., Lackner M. (2023). Characterization of Poly(3-hydroxybutyrate) (P3HB) from Alternative, Scalable (Waste) Feedstocks. Bioengineering.

[4] Philip S., Keshavarz T., Roy I. (2007). Polyhydroxyalkanoates: Biodegradable polymers with a range of applications. J. Chem. Technol. Biotechnol..

[5] Bugnicourt E., Cinelli P., Lazzeri A., Alvarez V. (2014). Polyhydroxyalkanoate (PHA): Review of synthesis, characteristics, processing and potential applications in packaging. Express Polym. Lett..

[6] Bugnicourt E., Cinelli P., Lazzeri A., Alvarez V.A. (2015). Main Characteristics, Properties, Improvements, and Market Data of Polyhydroxyalkanoates. Chapter 24 Handbook of Sustainable Polymers Processing and Applications.

[7] Vayshbeyn L.I., Mastalygina E.E., Olkhov A.A., Podzorova M.V. (2023). Poly(lactic acid)-Based Blends: A Comprehensive Review. Appl. Sci..

[8] Arrieta M.P., Samper M.D., Aldas M., López J. (2017). On the Use of PLA-PHB Blends for Sustainable Food Packaging Applications. Materials.

[9] Ikejima T., Cao A., Yoshie N., Inoue Y. (1998). Surface composition and biodegradability of poly(3-hydroxybutyric acid)/poly(vinyl alcohol) blend films. Polym. Degrad. Stab..

[10] Xing P., Ai X., Dong L., Feng Z. (1998). Miscibility and Crystallization of Poly(β-hydroxybutyrate)/Poly(vinyl acetate-*co*-vinyl alcohol) Blends. Macromolecules.

[11] Lotti N., Pizzoli M., Ceccorulli G., Scandola M. (1993). Binary blends of microbial poly(3-hydroxybutyrate) with polymethacrylates. Polymer.

[12] Garcia-Garcia D., Rayón E., Carbonell-Verdu A., Lopez-Martinez J., Balart R. (2017). Improvement of the compatibility between poly(3-hydroxybutyrate) and poly(ε-caprolactone) by reactive extrusion with dicumyl peroxide. Eur. Polym. J..

[13] Sukhanova A., Murzova A., Boyandin A., Kiselev E., Sukovatyi A., Kuzmin A., Shabanov A. (2020). Poly-3-hydroxybutyrate/chitosan composite films and nonwoven mats. Int. J. Biol. Macromol..

[14] Bharathiraja B., Sangeetha S. (2020). Polyhydroxyalkanoates and its composites for biomedical applications. Int. J. Biol. Macromol..

[15] Raza Z.A., Khalil S., Abid S. (2020). Recent progress in development and chemical modification of poly(hydroxybutyrate) based blends for potential medical applications. Int. J. Biol. Macromol..

[16] Conti D., Yoshida M., Pezzin S., Coelho L. (2006). Miscibility and crystallinity of poly(3-hydroxybutyrate)/poly(3-hydroxybutyrate-co-3-hydroxyvalerate) blends. Thermochim. Acta.

[17] Olkhov A.A., Markin V.S., Kosenko R.Y., Gol’dshtrakh M.A., Iordanskii A.L. (2015). Influence of the film forming procedure on the interaction in polyhydroxybutyrate-polyurethane blends. Russ. J. Appl. Chem..

[18] Wang S., Xiang H., Wang R., Peng C., Zhou Z., Zhu M. (2013). Morphology and properties of renewable poly(3-hydroxybutyrate-*co*-3-hydroxyvalerate) blends with thermoplastic polyurethane. Polym. Eng. Sci..

[19] Martínez-Abad A., González-Ausejo J., Lagarón J.M., Cabedo L. (2016). Biodegradable poly(3-hydroxybutyrate-co-3-hydroxyvalerate)/thermoplastic polyurethane blends with improved mechanical and barrier performance. Polym. Degrad. Stab..

[20] Panaitescu D.M., Melinte V., Frone A.N., Nicolae C.A., Gabor A.R., Capră L. (2022). Influence of Biobased Polyurethane Structure on Thermal and Mechanical Properties of Poly(3-hydroxybutyrate-co-3-hydroxyvalerate)−Polyurethane Blends. J. Polym. Environ..

[21] Frone A.N., Panaitescu D.M., Gabor A.R., Nicolae C.-A., Ghiurea M., Bradu C. (2024). Poly(3-hydroxybutyrate) Modified with Thermoplastic Polyurethane and Microfibrillated Cellulose: Hydrolytic Degradation and Thermal and Mechanical Properties. Polymers.

[22] Saha P., Khomlaem C., Aloui H., Kim B.S. (2021). Biodegradable Polyurethanes Based on Castor Oil and Poly (3-hydroxybutyrate). Polymers.

[23] Jin P., Pang A., Yang R., Guo X., He J., Zhai J. (2022). Study on Mechanical Properties of Polyurethane Cross-Linked P(E-co-T)/PEG Blended Polyether Elastomer. Polymers.

[24] Zarzyka I., Czerniecka-Kubicka A., Hęclik K., Dobrowolski L., Pyda M., Leś K., Walczak M., Białkowska A., Bakar M. (2021). Thermally stable biopolymer composites based on poly(3-hydroxybutyrate) modified with linear aliphatic polyurethanes–Preparation and properties. Acta Bioeng. Biomech..

[25] Zarzyka I., Czerniecka-Kubicka A., Hęclik K., Dobrowolski L., Krzykowska B., Białkowska A., Bakar M. (2022). Biobased poly(3-hydroxybutyrate acid) composites with addition of aliphatic polyurethane based on polypropylene glycols. Acta Bioeng. Biomech..

[26] Krzykowska B., Czerniecka-Kubicka A., Białkowska A., Bakar M., Hęclik K., Dobrowolski L., Longosz M., Zarzyka I. (2023). Polymer Biocompositions and Nanobiocomposites Based on P3HB with Polyurethane and Montmorillonite. Int. J. Mol. Sci..

[27] Yan X., Zhou W., Ma X., Sun B. (2020). Fabrication and characterization of poly(3-hydroxybutyrate-*co*-3-hydroxyhexanoate) modified with nano-montmorillonite biocomposite. e-Polymers.

[28] Panayotidou E., Baklavaridis A., Zuburtikudis I., Achilias D.S. (2014). Nanocomposites of poly(3-hydroxybutyrate)/organomodified montmorillonite: Effect of the nanofiller on the polymer’s biodegradation. J. Appl. Polym. Sci..

[29] Chinthalapalli S., Soundiraraju B., Siripothu S., Dash S.S. (2023). Insights into the Improved Gas Barrier Properties of Polyurethane–Clay Nanocomposite-Coated Nylon: A Solid-State NMR Study. ACS Omega.

[30] Pérez G., Jin A., del Valle L.J., Fontdecaba E., Puiggalí J. (2024). Ultrasonic Molding of Poly(3-hydroxybutyrate) and Its Clay Nanocomposites: Efficient Microspecimens Production with Minimal Material Loss and Degradation. Appl. Sci..

[31] Bangar S.P., Whiteside W.S., Chaudhary V., Akhila P.P., Sunooj K.V. (2023). Recent functionality developments in Montmorillonite as a nanofiller in food packaging. Trends Food Sci. Technol..

[32] Kalendova A., Kupkova J., Urbaskova M., Merinska D. (2024). Applications of Clays in Nanocomposites and Ceramics. Minerals.

[33] Hassen J.H., Abdalkadir H.K., Abed S.F. (2023). An overview of medical applications of montmorillonite clay. J. Med. Sci..

[34] Bee S.-L., Abdullah M.A.A., Bee S.-T., Sin L.T., Rahmat A.R. (2018). Polymer nanocomposites based on silylated-montmorillonite: A review. Prog. Polym. Sci..

[35] Gómez M., Palza H., Quijada R. (2016). Influence of Organically-Modified Montmorillonite and Synthesized Layered Silica Nanoparticles on the Properties of Polypropylene and Polyamide-6 Nanocomposites. Polymers.

[36] Chang M.-K., Lee H.-C. (2015). Effects of montmorillonite and compatibilizer on the mechanical and thermal properties of dispersing intercalated PMMA nanocomposites. Int. Commun. Heat Mass Transf..

[37] Xiong J., Zheng Z., Jiang H., Ye S., Wang X. (2007). Reinforcement of polyurethane composites with an organically modified montmorillonite. Compos. Part A Appl. Sci. Manuf..

[38] Soares Silva L.C., Busto R.V., Camani P.H., Zanata L., Gomes Coelho L.H., Benassi R.F., dos Santos Rosa D. (2021). Influence of Montmorillonite and Clinoptilolite on the Properties of Starch/Minerals Biocomposites and Their Effect on Aquatic Environments. J. Polym. Environ..

[39] Reddy M.M., Deighton M., Bhattacharya S., Parthasarathy R. (2009). Biodegradation of montmorillonite filled oxo-biodegradable polyethylene. J. Appl. Polym. Sci..

[40] Yussuf A.A., Al-Saleh M.A., Al-Samhan M.M., Al-Enezi S.T., Al-Banna A.H., Abraham G. (2017). Investigation of Polypropylene-Montmorillonite Clay Nanocomposite Films Containing a Pro-degradant Additive. J. Polym. Environ..

[41] Zarzyka I., Krzykowska B., Hęclik K., Frącz W., Janowski G., Bąk Ł., Klepka T., Bieniaś J., Ostapiuk M., Tor-Świątek A. (2024). Modification of Poly(3-Hydroxybutyrate) with a Linear Polyurethane Modifier and Organic Nanofiller—Preparation and Structure–Property Relationship. Materials.

[42] (2020). Plastics. Determination of Mechanical Properties in Static Tension. Part 1: General Rules.

[43] (2019). Plastics. Determination of Bending Properties.

[44] (2012). Plastics–Determination of the Ultimate Aerobic Biodegradability of Plastic Materials in Soil by Measuring the Oxygen Demand in a Respirometer or the Amount of Carbon Dioxide Evolved.

[45] (2019). Soil Quality–Determination of the Water-Retention Characteristic–Laboratory Methods.

[46] (2021). Soil Quality–Determination of pH.

[47] Alsaadi M., Erkliğ A. (2019). Effects of clay and silica nanoparticles on the Charpy impact resistance of a carbon/aramid fiber reinforced epoxy composite. Mater. Test..

[48] Liang J. (2018). Impact fracture behavior and morphology of polypropylene/graphene nanoplatelets composites. Polym. Compos..

[49] Demircan Ö., Kadıoğlu K., Çolak P., Günaydın E., Doğu M., Topalömer N., Eskizeybek V. (2020). Compression after Impact and Charpy Impact Characterizations of Glass Fiber/Epoxy/MWCNT Composites. Fibers Polym..

[50] Kumar G.N., Kumar C.S., Rao G.S. (2019). An experimental investigation on mechanical properties of hybrid polymer nanocomposites. Mater. Today Proc..

[51] Champa-Bujaico E., Díez-Pascual A.M., Redondo A.L., Garcia-Diaz P. (2024). Optimization of mechanical properties of multiscale hybrid polymer nanocomposites: A combination of experimental and machine learning techniques. Compos. Part B Eng..

[52] Botana A., Mollo M., Eisenberg P., Sanchez R.M.T. (2010). Effect of modified montmorillonite on biodegradable PHB nanocomposites. Appl. Clay Sci..

[53] Panaitescu D.M., Lupescu I., Frone A.N., Chiulan I., Nicolae C.A., Tofan V., Stefaniu A., Somoghi R., Trusca R. (2017). Medium chain-length polyhydroxyalkanoate copolymer modified by bacterial cellulose for medical devices. Biomacromolecules.

[54] Freier T. (2006). Biopolyesers in Tissue Engineering Applications. Polymers for Regenerative Medicine.

[55] Ikejima T., Inoue Y. (2000). Crystallization behavior and environmental biodegradability of the blend films of poly(3-hydroxybutyric acid) with chitin and chitosan. Carbohydr. Polym..

[56] Almustafa W., Schubert D.W., Grishchuk S., Sebastian J., Grun G. (2024). Chemical Synthesis of Atactic Poly-3-hydroxybutyrate (a-P3HB) by Self-Polycondensation: Catalyst Screening and Characterization. Polymers.

[57] Lejardi A., Etxeberria A., Meaurio E., Sarasua J.-R. (2012). Novel poly(vinyl alcohol)-g-poly(hydroxy acid) copolymers: Synthesis and characterization. Polymer.

[58] Brtnicky M., Pecina V., Kucerik J., Hammerschmiedt T., Mustafa A., Kintl A., Sera J., Koutny M., Baltazar T., Holatko J. (2024). Biodegradation of poly-3-hydroxybutyrate after soil inoculation with microbial consortium: Soil microbiome and plant responses to the changed environment. Sci. Total. Environ..

[59] Kim J., Gupta N.S., Bezek L.B., Linn J., Bejagam K.K., Banerjee S., Dumont J.H., Nam S.Y., Kang H.W., Park C.H. (2023). Biodegradation Studies of Polyhydroxybutyrate and Polyhydroxybutyrate-*co*-Polyhydroxyvalerate Films in Soil. Int. J. Mol. Sci..

[60] Singh K., Pralay M., Mittal V. (2011). 12 Biodegradable nanocomposites based on poly(hydroxyalkanoates). Nanocomposites with Biodegradable Polymers: Synthesis, Properties, and Future Perspectives, Monographs on the Physics and Chemistry of Materials.

[61] Kotal M., Bhowmick A.K. (2015). Polymer nanocomposites from modified clays: Recent advances and challenges. Prog. Polym. Sci..

[62] Nikolaidis A.K., Achilias D.S., Karayannidis G.P. (2011). Synthesis and Characterization of PMMA/Organomodified Montmorillonite Nanocomposites Prepared by in Situ Bulk Polymerization. Ind. Eng. Chem. Res..

[63] Białkowska A., Krzykowska B., Zarzyka I., Bakar M., Sedlařík V., Kovářová M., Czerniecka-Kubicka A. (2023). Polymer/Layered Clay/Polyurethane Nanocomposites: P3HB Hybrid Nanobiocomposites—Preparation and Properties Evaluation. Nanomaterials.

[64] Dayma N., Satapathy B.K. (2012). Microstructural correlations to micromechanical properties of polyamide-6/low density polyethylene-grafted-maleic anhydride/nanoclay ternary nanocomposites. Mater. Des..

[65] Loyens W., Jannasch P., Maurer F.H.J. (2005). Effect of clay modifier and matrix molar mass on the structure and properties of poly(ethylene oxide)/Cloisite nanocomposites via melt-compounding. Polymer.

